# Effects of different oral care scrubs on ventilator-associated pneumonia prevention for machinery ventilates patient: A protocol for systematic review, evidence mapping, and network meta-analysis

**DOI:** 10.1097/MD.0000000000014923

**Published:** 2019-03-22

**Authors:** Hua-ping Wei, Kelu Yang

**Affiliations:** a Department of Nursing, The First Hospital of Lanzhou University; b Evidence-Based Nursing Center, School of Nursing, Lanzhou University, Lanzhou City, Gansu Province, China.

**Keywords:** evidence mapping, network meta-analysis, oral care, systematic review, ventilator-associated pneumonia

## Abstract

**Background:**

Ventilator-associated pneumonia (VAP) is defined as pneumonia develops in intensive care unit (ICU) patients who have been mechanically ventilated for at least 48 hours. Implementing effective oral car could reduce the incidence of VAP. However, previous studies on scrubs in oral care have failed to suggest which the best choice. Therefore, this protocol proposes to perform a network meta-analysis to evaluate the effectiveness of different oral care scrubs in preventing VAP.

**Methods:**

We are going to search the electronic databases: Cochrane Oral Health's Trials Register, CENTRAL, MEDLINE, EMBASE, CINAHL, and Chinese Biomedical Literature Database. Study selection and data collection will be performed independently by 2 reviewers. Cochrane Risk of Bias tool will be used to assess the risk of bias of included studies. Odds ratio (OR) and 95% confidence intervals (CIs) will be used to assess the incidence rate of VAP in critical patients. The evidence mapping (EM) method will be introduce as a tool intended to complement the conventional systematic review (SR) and is suitable for this issue, at the same time, R software will be used for representing the outcome of EM–SR. We shall assess the heterogeneity on the bias of the magnitude of heterogeneity variance parameter (*I*^2^ or Cochrane *Q*). We are also going to conduct subgroup analysis and sensitivity analysis if needed. The application of Stata and R software will be performed the calculations.

**Results:**

The results of this study will be submitted to a peer-reviewed journal for publication.

**Conclusion:**

This network meta-analysis will provide comprehensive evidence of different scrubs in oral care for preventing VAP.

**PROSPERO registration number:**

CRD42018117019.

## 1 Introduction

Hospital-acquired pneumonia (HAP) is an infection of the pulmonary parenchyma caused by pathogenic bacterium that are present in hospital settings, which is the second most common nosocomial infection and the leading cause of death in hospital-acquired infections in critical patients.^[1]^ Ventilator-associated pneumonia (VAP) is one of the forms of HAP, which develops in intensive care unit (ICU) patients who have been mechanically ventilated for at least 48 hours.^[2]^ The epidemiological study of International Nosocomial Infection Control Consortium (INICC)^[3]^ report an incidence of VAP per 1000 mechanical ventilator-days is 13.1 in the medical surgical ICUs and 9.02 in the NICUs. Moreover, the evidences have showed that the cost of VAP was massive, calculating up to $20,000 in Europe and the United States.^[4,5]^ Therefore, it is significantly important for the clinicians to well reduce the incidence of VAP and is also the strategy of improving ICU care.

It is acknowledged that plentiful risk factors could lead to VAP, which contained the sedation and curarization, heart and lung-associated disease, regurgitation and aspiration, and so on.^[6,7]^ Several studies have indicated that there were some pathologic changes in oral cavity in ICU patients, including oral mucosal lesions, periodontal disease relief, dryness of the lips and mucous membranes, fungal infections, increased biofilm on the oral surface, etc. The micro-aspiration of oropharynx colonization agents is a significant cause of most VAP cases.^[8–10]^ Patients who receive mechanical ventilation must keep their mouths open and are not able to chew, resulting in reducing saliva flow and mucous membranes dryness.^[11,12]^

Oral care is a basic care activity for critical patients, making them comfort and relief. And the necessity of oral care in ICU for ventilation patients has already been discussed and confirmed.^[13,14]^ A published Practice Alert demonstrated that a comprehensive oral hygiene program should be set for critical patients with or without ventilation.^[15]^ Furthermore, the Ventilator Bundle was published by Institute for Healthcare Improvement (IHI)^[16]^ in 2012 demonstrated the five aspects to prevent VAP, mainly including the item of raising the head of the bed to between 30° and 45°; the assessment of “sedative interruption” and daily extubation preparation, the prevention of peptic ulcer disease; the deep venous thrombosis prophylaxis (unless contraindicated), and oral care using chlorhexidine daily. Whether continuous or intermittent subglottic secretion drainage is superior for preventing VAP has already been discussed.^[17]^ As for the tools for oral care, there had not been firmed recommended evidence. A Cochrane systematic review showed that there was no difference in the rate of VAP comparing tooth brushing and no brushing, as same as the outcomes on mortality and duration of ventilation.^[18,19]^ But other systematic review concluded that using tooth brushing could find a trend toward lower VAP rate.^[20]^ However, the published systematic reviews on these topics included inadequate numbers of studies, which ranged from 4 to 6 records, and it is obviously difficult to conclude comprehensive and robust evidence on the oral care for patients with mechanical ventilation, even likely lead to the selective bias of the result due to the insufficient of the interventions and including studies. What's more, there are some clinical trials compared manual toothbrush with cotton ball,^[21]^ toothette,^[22,23]^ gauze,^[24]^ or electronic toothbrush.^[25]^ Unfortunately, it is hard to tell which the best choice for the clinic working is. In addition, toothbrush is beneficial to removing plaque, and the electric toothbrush showed the better ability than manual toothbrush.^[26]^ When it comes to the preferred toothbrush tool for oral care, it still puzzle the clinicians.

In terms of methodology, because there are >2 brushing scrubs, it is necessary to use systematic review (SR), which helps to cope with the rapid increase of clinical literature,^[27]^ and network meta-analysis (NMA), a promotion of pairwise meta-analysis allows the simultaneous evaluation of the relative effectiveness of several interventions through a randomized clinical trial (RCT) network, which has been increasing worldwide under the leadership of western countries.^[28]^ As supplementary, evidence mapping (EM), a method of evidence summary,^[29–31]^ could be used to summarize the basic characteristics and results of the included studies, as well as to present the overall depth and breadth of the evidence.

The aim of this study is to perform an evidence mapping and network meta-analysis comparing the effect of different scrubs used in oral care for the prevention of VAP based on existing randomized controlled trials (RCTs).

## 2 Methods

### 2.1 Study registration

The content of this protocol follows the PRISMA Protocols (PRISMA-P) recommendations.^[32]^ This review has been registered on the International Prospective Register of Systematic Reviews (PROSPERO),^[33]^ with the registration number CRD42018117019. If protocol amendments occur, the dates, changes, and rationales will be tracked in PROSPERO.

### 2.2 Ethics and dissemination

Because this study is not a clinical study, ethical approval is not required.

### 2.3 Data sources and search strategy

Search strategies will be performed on six electronic databases. The databases consulted are: Cochrane Oral Health's Trials Register, Cochrane Central Register of Controlled Trials (CENTRAL), PubMed, EMBASE, Cumulative Index to Nursing and Allied Health Literature (CINAHL), and Chinese Biomedical Literature Database. The MeSH search and text word search will be used with the terms related to critical illness, critical care, oral hygiene, mouthwashes, and randomized controlled trial. All the references lists of the included studies will be checked to identify any additional studies. The first or corresponding authors of the included studies, other experts in the field, and manufacturers of oral hygiene products shall be contacted to request unpublished relevant information.

What the specific search strategy will be (taking PubMed as an example) is shown in Table 1. The strategy will be modified for other databases use if necessary.

**Table 1:**
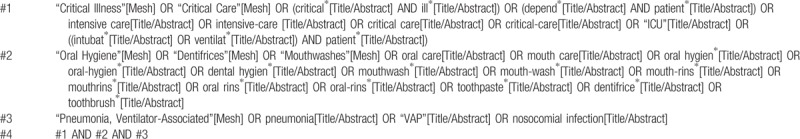
Searching strategy in PubMed.

### 2.4 Criteria for including studies in this review

#### 2.4.1 Types of participants

Patients who are 18 years or older received mechanically ventilated hospitalized in ICU will be included. Patients already diagnosed with VAP, edentulous patients, studies involving children, and pregnant patients will be excluded.

#### 2.4.2 Types of interventions

Oral care includes different tools, such as using manual toothbrush, electric toothbrush, toothette, cotton ball, gauze, or others.

#### 2.4.3 Types of outcome

##### 2.4.3.1 Primary outcome

Incidence of VAP: VAP is defined as the pneumonia that develops in intensive care unit (ICU) patients who have been mechanically ventilated for at least 48 hours.^[2]^

##### 2.4.3.2 Secondary outcome

a. Mortality rate: either ICU mortality if these data were available, or 30-day mortality.
b. Duration of mechanical ventilation: We define the duration of mechanical ventilation as the time period from receiving mechanical ventilation to weaning it.
c. Duration of ICU stay: we define the duration of ICU stay as the time period from the timing of admission in ICU to discharge from ICU, according to the record in the hospital information system.
d. Oral health indices: such as gingival index, plaque index, bleeding index, periodontal index, etc.
e. Adverse outcomes resulting from the interventions: such as intracranial pressure (ICP) increasing during the oral care in neurosurgical patients.^[34]^

#### 2.4.4 Types of studies

RCTs of prevention of VAP will be included to pool and review in this study. Nonrandomized controlled trials, observational studies, qualitative studies, and laboratory studies will be excluded.

### 2.5 Data extraction and quality assessment

#### 2.5.1 Selection of studies

Literature search records will be imported into EndNote X8 literature management software (Thomson Reuters [Scientific] LLC, Philadelphia, PA). Two reviewers are about to screen the titles and abstracts of retrieved studies to identify potentially eligible studies. Then they will select full-text of potentially eligible studies and determine study for inclusion or exclusion. All the works above will be done independently. Any disagreement will be resolved by the third part. The selection process will be summarized according to PRISMA flow diagram.

#### 2.5.2 Data extraction and management

First, predesigned data extraction form is to be designed by our team. Then, 1 to 5 included studies will be pre-extracted. If necessary, the forms shall be continually modified until the final data extraction form complete. Two reviewers (YG, KLY) will independently extract data from each included study. Different opinions will be resolved through discussion or consult the third part (JHT).

The following items will be extracted: general characteristics of the study: author, year of publication, country where the study was performed, funding, study duration, contact details of the authors and identifier; specific trial characteristics: sequence generation, allocation sequence concealment, blinding, incomplete outcome data, and selective outcome reporting, etc., and present them in the table of characteristics of included studies; participants: total number, setting, age, sex, country, sociodemographic details (e.g., education level; health insurance), diagnostic criteria for VAP, and the presence of comorbid conditions; interventions: we are going to collect details of all experimental and control interventions, such as dosages for drugs used, the scrubs for oral hygiene care, timing and duration of the oral care procedures, systemic antibiotic use, and other interventions that may affect the outcomes; outcomes: we are going to collect the incidence of VAP or other respiratory diseases, mortality (directly and indirectly attributable), duration of mechanical ventilation, duration of ICU stay, oral health indices, and adverse outcomes resulting from the interventions, etc.; other results: we are also going to collect key conclusions, comments, and any explanations provided for unexpected findings by the study authors. We shall contact the lead authors of included studies if there are issues to be clarified.

#### 2.5.3 Risk of bias assessment

Two reviewers will evaluate the risk of bias of the included studies by using the Cochrane Risk of Bias tool.^[35]^ We are going to complete a risk of bias table for all the included study. Each included study will be assigned a level of risk of bias (high risk, unclear risk, low risk) for each domain. Any disagreement will be resolved through discussion or consult the third part.

#### 2.5.4 Management of missing data

If there is missing data in several included studies, we shall contact the corresponding author to request any inadequate and missing data by E-mail. If the data are still not available, we are about to perform data synthesis through existing information and address the potential impact of missing data on the pooled results in the discussion parts.

#### 2.5.5 Data synthesis

We are going to provide a narrative synthesis of the findings from the included studies, the type of intervention, the target population characteristics, the type of outcome, and the intervention content. We shall also provide summaries of the intervention effects for each study by calculating odds ratios (OR) for dichotomous outcomes. The odds ratio and 95% CIs will be used to assess the incidence rate of ventilator-associated pneumonia in critical patients. We will apply Stata 15.0 and R (version 3.4.1; R Foundation for Statistical Computing, Vienna, Austria) software to perform the calculations.

#### 2.5.6 Assessment of heterogeneity

Assessment of heterogeneity between the included studies will be conducted to evaluate the feasibility of network meta-analysis. We shall assess the heterogeneity on the bias of the magnitude of heterogeneity variance parameter (*I*^2^ or Cochrane *Q*). If the *P* > .05 for *Q* test or *I*^2^ <50% for *I*^2^ test, which suggests there is no statistical heterogeneity, then the Mantel–Haenszel fixed effect model will be employed, whereas the *P* < .05 for *Q* test or *I*^2^ > 50% for *I*^2^ test, we will explore sources of heterogeneity by subgroup analysis and meta-regression. If no clinical heterogeneity was identified, the Mantel–Haenszel random effects model will be used. A node splitting method will be used to examine the inconsistency between direct and indirect comparisons when a loop connecting 3 arms exists.

#### 2.5.7 Subgroup analysis

If sufficient evidence is available, we are going to conduct subgroup analyses to explore the difference between men and women; smoking and non-smoking; with and without rising, etc.

#### 2.5.8 Sensitivity analysis

Sensitivity analysis will be conducted by changing the grouping of the different intervention measures to see the influence of the individual dataset on the pooled ORs. The results will not be substantially changed when any study is excluded if the pooled ORs are robust.

#### 2.5.9 Publication bias

Begg funnel plot will be conducted to evaluate the publication bias of the included studies. If publication bias is detected *P* < .5 is considered statistically significant, we will perform the trim and fill test for further analysis.

#### 2.5.10 Quality of evidence

The results of the main outcomes (primary outcomes, the duration of mechanical ventilation, and ICU stay) will be summarized in the summary of findings tables. We are going to adopt The Grading of Recommendations Assessment, Development, and Evaluation (GRADE)^[36]^ approach to assess the quality of evidence of the pooled studies. Limitations of the study, inconsistencies, indirect evidence, inaccuracies, and publication bias will be considered. Levels of evidence quality will be classified into 4 levels: high, moderate, low, or very low.

#### 2.5.11 Evidence mapping

Until now, there has been no official standard methodology developed for an EM.^[37]^ Therefore, we consider to complete EM via R (version 3.4.1; R Foundation for Statistical Computing, Vienna, Austria) software. The presentation form of EM will be a bubble plot which display information on 4 dimensions: the sample size of each included study (bubble size); assessment of risk of bias (bubble label); the scrubs used in the included studies (*y*-axis); and the rating of conclusions (*x*-axis).

## 3 Discussion

Oral care for the prevention of VAP has been widely used in the ICU, which effectiveness has been confirmed, but the details of the operational process are still being worthy of discussion. So far, there is still no best recommendation for the scrubs used in oral care. This provided the first motivation for this study. In addition, the evaluation of >2 inventions by traditional meta-analysis is currently limited and difficult. It is unclear which oral scrub is best for oral care and there are no enough head-to-head RCTs to compare the efficacy of the different scrubs. This study will compare different scrubs using a network meta-analysis to determine the better method and present an evidence mapping as supplement to help make clinical decisions.

## Author contributions

**Conceptualization:** Huaping Wei, Kelu Yang.

**Funding acquisition:** Huaping Wei, Kelu Yang.

**Methodology:** Huaping Wei, Kelu Yang.

**Project administration:** Huaping Wei, Kelu Yang.

**Writing – original draft:** Huaping Wei, Kelu Yang.

**Writing – review & editing:** Huaping Wei, Kelu Yang.
